# Disgust and Self-Disgust in Eating Disorders: A Systematic Review and Meta-analysis

**DOI:** 10.1192/j.eurpsy.2023.916

**Published:** 2023-07-19

**Authors:** S. Bektas, J. L. Keeler, H. Mutwalli, H. Himmerich, J. Treasure

**Affiliations:** 1 Section of Eating Disorders, Department of Psychological Medicine, Institute of Psychiatry, Psychology & Neuroscience, King’s College London, London, United Kingdom; 2 Department of Psychology, Hacettepe University, Ankara, Türkiye; 3 Section of Eating Disorders, Department of Psychological Medicine, Institute of Psychiatry, Psychology and Neuroscience, King’s College London, London, United Kingdom; 4 Department of Clinical Nutrition, College of Applied Medical Sciences, Imam Abdulrahman Bin Faisal University, Dammam, Saudi Arabia; 5 Eating Disorders Unit, Bethlem Royal Hospital, South London and Maudsley NHS Foundation Trust (SLaM), Maudsley Hospital; 6 Eating Disorders Unit, Bethlem Royal Hospital, South London and Maudsley NHS Foundation Trust (SLaM), Maudsley Hospital, London, United Kingdom

## Abstract

**Introduction:**

Disgust and self-disgust are aversive emotions which are often encountered in people with eating disorders.

**Objectives:**

The aim of this systematic review is to conduct a synthesis of studies that have measured aspects of disgust and self-disgust in people with EDs.

**Methods:**

We conducted a systematic review and meta-analysis of disgust and self-disgust in people with eating disorders using Preferred Reporting Items for Systematic Reviews and Meta-Analyses (PRISMA) guidelines. The systematic review of the literature revealed 52 original research papers.

**Results:**

There was substantial heterogeneity regarding the research question and outcomes. However, we found 5 articles on disgust elicited by food images, 10 studies on generic disgust sensitivity, and 4 studies on self-disgust, and we proceeded to a meta-analytic approach on these studies. We found that women with eating disorders have significantly higher momentary disgust feelings in response to food images (1.32; 95% CI 1.05, 1.59), higher generic disgust sensitivity (0.49; 95% CI 0.24, 0.71), and higher self-disgust (1.90; 95% CI 1.51, 2.29) compared with healthy controls.

**Conclusions:**

These findings indicate the potential clinical relevance of disgust and self-disgust in the treatment of eating disorders.

**Disclosure of Interest:**

None Declared

